# Cell-Fusing Agent Virus Reduces Arbovirus Dissemination in Aedes aegypti Mosquitoes *In Vivo*

**DOI:** 10.1128/JVI.00705-19

**Published:** 2019-08-28

**Authors:** Artem Baidaliuk, Elliott F. Miot, Sebastian Lequime, Isabelle Moltini-Conclois, Fanny Delaigue, Stéphanie Dabo, Laura B. Dickson, Fabien Aubry, Sarah H. Merkling, Van-Mai Cao-Lormeau, Louis Lambrechts

**Affiliations:** a Insect-Virus Interactions Unit, UMR2000, CNRS, Institut Pasteur, Paris, France; b Collège doctoral, Sorbonne Université, Paris, France; c Medical Entomology and Vector-Borne Disease Unit, Institut Pasteur du Laos, Vientiane, Lao PDR; d Unit of Emerging Infectious Diseases, Institut Louis Malardé, Papeete, Tahiti, French Polynesia; University of Texas Southwestern Medical Center

**Keywords:** *Aedes aegypti*, arbovirus, coinfection, insect-specific virus, superinfection, viral interference

## Abstract

Aedes aegypti mosquitoes are the main vectors of arthropod-borne viruses (arboviruses) of public health significance, such as the flaviviruses dengue virus (DENV) and Zika virus (ZIKV). Mosquitoes are also the natural hosts of a wide range of viruses that are insect specific, raising the question of their influence on arbovirus transmission in nature. Cell-fusing agent virus (CFAV) was the first described insect-specific flavivirus, initially discovered in an A. aegypti cell line and subsequently detected in natural A. aegypti populations. It was recently shown that DENV and the CFAV strain isolated from the A. aegypti cell line have mutually beneficial interactions in mosquito cells in culture. However, whether natural strains of CFAV and DENV interact in live mosquitoes is unknown. Using a wild-type CFAV isolate recently derived from Thai A. aegypti mosquitoes, we found that CFAV negatively interferes with both DENV type 1 and ZIKV *in vitro* and *in vivo*. For both arboviruses, prior infection by CFAV reduced the dissemination titer in mosquito head tissues. Our results indicate that the interactions observed between arboviruses and the CFAV strain derived from the cell line might not be a relevant model of the viral interference that we observed *in vivo*. Overall, our study supports the hypothesis that insect-specific flaviviruses may contribute to reduce the transmission of human-pathogenic flaviviruses.

**IMPORTANCE** The mosquito Aedes aegypti carries several arthropod-borne viruses (arboviruses) that are pathogenic to humans, including dengue and Zika viruses. Interestingly, A. aegypti is also naturally infected with insect-only viruses, such as cell-fusing agent virus. Although interactions between cell-fusing agent virus and dengue virus have been documented in mosquito cells in culture, whether wild strains of cell-fusing agent virus interfere with arbovirus transmission by live mosquitoes was unknown. We used an experimental approach to demonstrate that cell-fusing agent virus infection reduces the propagation of dengue and Zika viruses in A. aegypti mosquitoes. These results support the idea that insect-only viruses in nature can modulate the ability of mosquitoes to carry arboviruses of medical significance and that they could possibly be manipulated to reduce arbovirus transmission.

## Abstract (precis)

The mosquito Aedes aegypti carries several arthropod-borne viruses (arboviruses) that are pathogenic to humans, including dengue and Zika viruses. Interestingly, A. aegypti is also naturally infected with insect-only viruses, such as cell-fusing agent virus. Although interactions between cell-fusing agent virus and dengue virus have been documented in mosquito cells in culture, whether wild strains of cell-fusing agent virus interfere with arbovirus transmission by live mosquitoes was unknown. We used an experimental approach to demonstrate that cell-fusing agent virus infection reduces the propagation of dengue and Zika viruses in A. aegypti mosquitoes. These results support the idea that insect-only viruses in nature can modulate the ability of mosquitoes to carry arboviruses of medical significance and that they could possibly be manipulated to reduce arbovirus transmission.

## INTRODUCTION

Aedes aegypti mosquitoes are major vectors of multiple medically important arthropod-borne viruses (arboviruses), such as dengue virus (DENV) and Zika virus (ZIKV) (*Flavivirus*, *Flaviviridae*). There are an estimated 390 million DENV infections every year, of which 96 million result in clinical symptoms, such as fever, headache, joint pain, and rash (1). ZIKV was a little-known virus until it caused several major outbreaks in the Pacific and in Latin America in recent years. Even though in most cases ZIKV infection in humans is asymptomatic, it can cause Guillain-Barré syndrome and congenital microcephaly (2, 3). Interestingly, A. aegypti mosquitoes are also naturally infected with other viruses of the genus *Flavivirus* that are capable of replication only in insect cells (4). Cell-fusing agent virus (CFAV; *Flavivirus*, *Flaviviridae*) was the first described insect-specific flavivirus (ISF) infecting an A. aegypti cell line (5). It was named after its cytopathic effect (CPE) in Aedes albopictus cells: fusion of cells. After its isolation in 1975 and genome sequencing in 1992, CFAV was detected and/or isolated mainly from *Aedes* species mosquitoes in regions where dengue is endemic (6–12). Since the discovery of CFAV more than 4 decades ago, a large number of other ISFs have been reported in various mosquito species. ISFs circulate in natural mosquito populations (7), they are transmitted predominantly vertically (13), and they can also be experimentally inoculated into mosquitoes by intrathoracic (IT) injection (14).

The widespread occurrence of ISFs in natural mosquito populations and their close phylogenetic relationship with human-pathogenic flaviviruses have raised the question of their potential influence on arbovirus transmission by mosquitoes (4). Understanding the nature of interactions between ISFs and arboviruses not only contributes to elucidation of the natural drivers of arbovirus transmission but also supports the potential use of ISFs as disease control agents against arboviruses (15). Moreover, there is a need to evaluate the potential effect of ISFs on the efficacy of novel disease control strategies based on the release of mosquitoes artificially infected with *Wolbachia* (16, 17). In the last few years, several studies have examined interactions between arboviruses and insect-only viruses, including ISFs, and this body of work has been reviewed extensively elsewhere (4, 15, 18–21). With only a few exceptions (22–24), however, most of these studies have been conducted *in vitro*. In particular, it was recently shown that CFAV and DENV type 2 (DENV-2) mutually interact in the A. aegypti cell line Aa20 (25). In these experiments, Aa20 cells were coinfected with DENV-2 and the CFAV strain derived from the persistently infected A. aegypti cell line Aag2. CFAV infection enhanced DENV-2 replication at days 1 and 3 after DENV-2 inoculation, although DENV-2 replication was inhibited at day 5 (25). Reciprocally, it was observed that DENV-2 promoted CFAV replication at days 1 and 3 but not at day 5 (25). Another study reported inhibition of ZIKV replication by Phasi Charoen-like virus (PCLV; *Phasivirus*, *Bunyaviridae*) in the A. albopictus cell line Aa23, which is persistently infected with CFAV, but the effect of PCLV in the absence of CFAV was not investigated (26).

While these earlier *in vitro* studies have contributed to improve our understanding of the complexity of interactions of CFAV with arboviruses, whether the results obtained in cell culture can be extrapolated to mosquitoes *in vivo* is currently unknown. Moreover, using the CFAV strain derived from the A. aegypti cell line Aag2, which had been persistently infecting this cell line for decades (5, 27, 28), could have been misleading. Indeed, the Aag2 cell-derived CFAV strain is genetically divergent from all other known CFAV strains (11, 29). Of note, the codon-overlapping gene named *fifo*, a −1 open reading frame conserved among ISFs, is disrupted by several premature stop codons in the Aag2 cell-derived CFAV strain (29). Although the function of the *fifo*-encoded protein is unknown, its evolutionary conservation across other ISFs suggests an important role *in vivo*.

Here, we assessed the ability of a wild-type strain of CFAV to interact with DENV type 1 (DENV-1) and ZIKV both *in vitro* and *in vivo*. We first evaluated the ability of the wild-type CFAV strain to interfere with DENV-1 and ZIKV replication in the CFAV-free A. albopictus cell line C6/36, followed by *in vivo* experiments in CFAV-free A. aegypti mosquitoes. Overall, our study indicated that CFAV negatively interferes with both DENV-1 and ZIKV *in vitro* and *in vivo*, which could contribute to reduce arbovirus transmission by mosquitoes in nature.

## RESULTS

**FIG 1:**
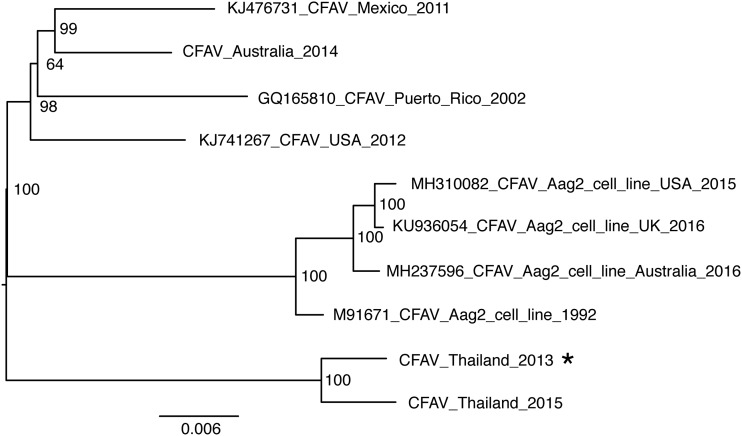
Phylogenetic relationships among CFAV strains. The tree represents the consensus of 1,000 bootstrap maximum likelihood trees based on the nucleotide alignment of full or nearly full genome sequences of CFAV and a GTR+F+I substitution model. The node support values represent bootstrap proportions. The tree is midpoint rooted, and the scale bar represents the number of nucleotide substitutions per site. The asterisk indicates the newly isolated CFAV strain from this study. GenBank accession numbers are indicated at the beginning of the leaf labels. “Aag2_cell_line” indicates that the virus is derived from the Aag2 cell line, as opposed to live mosquitoes. The country name indicates the geographical origin of mosquitoes from which the virus was isolated or the country where the Aag2 cell line was maintained prior to CFAV sequencing. The year at the end of each name represents the year of CFAV isolation, except for M91671_CFAV_Aag2_cell_line_1992, where 1992 is the year of sequencing since the year of isolation is unknown. Sequences without accession numbers (CFAV_Australia_2014 and CFAV_Thailand_2015) were obtained directly from the authors (12).

We isolated a CFAV strain from A. aegypti adults at the 4th generation of a colony originating from Kamphaeng Phet, Thailand, in 2013. The virus was isolated and amplified in the A. albopictus C6/36 cell line, and its full genome sequence was obtained by deep sequencing of a cDNA library and rapid amplification of cDNA ends (RACE). Phylogenetic analysis against the backdrop of currently available full or nearly full genome sequences of CFAV (Fig. 1) showed that this new CFAV strain is most closely related to another CFAV sequence detected in 2015 in Thai mosquitoes (12). The *fifo* −1 open reading frame conserved among ISFs (with the exception of the Aag2 cell-derived strain, where it is disrupted by premature stop codons) is intact in the new wild-type CFAV genome. Our phylogenetic analysis also confirmed that all published CFAV sequences obtained from the Aag2 cell line form a distant clade from the rest of the tree (Fig. 1).

**FIG 2:**
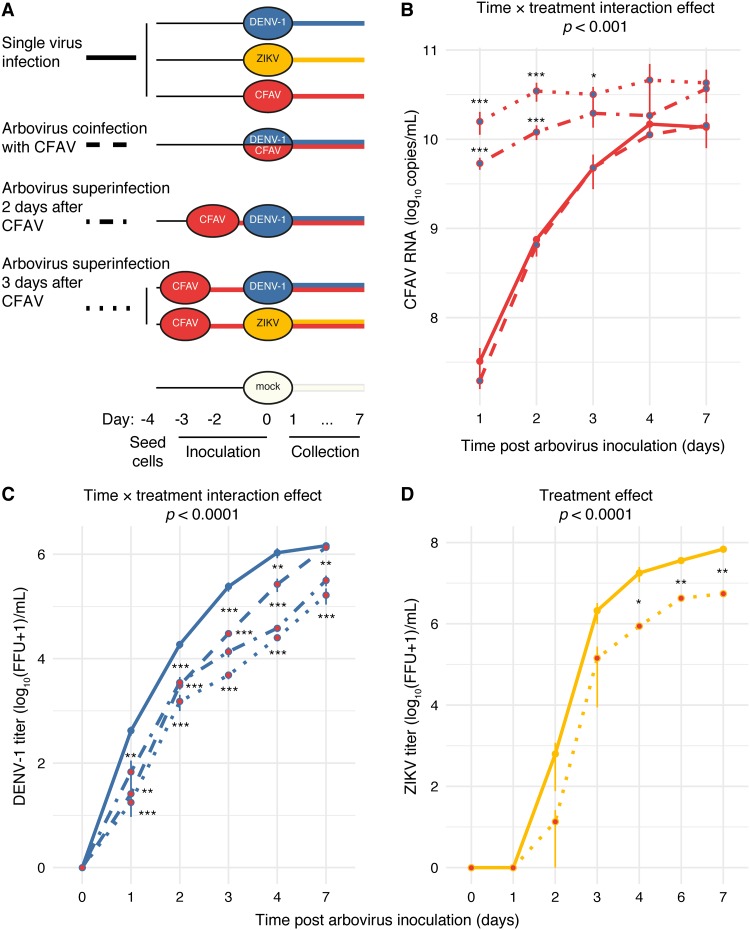
CFAV inhibits DENV-1 and ZIKV replication *in vitro*. (A) The schematic summarizes the experimental design of the *in vitro* studies and shows the different treatments and controls with their inoculation time line. C6/36 cells were inoculated with DENV at 0, 2, or 3 days after CFAV infection or with ZIKV at 3 days after CFAV infection. (B) Growth curves represent CFAV RNA levels on a log_10_ scale measured by absolute RT-qPCR in the presence (blue filled dots) or absence (red filled dots) of DENV-1. (C and D) Infectious titers of DENV-1 (C) and ZIKV (D) over time in log_10_-transformed focus-forming units (FFUs) per milliliter of C6/36 cell culture supernatant in the presence (red filled dots) or absence (blue filled dots for DENV-1 or yellow filled dots for ZIKV) of CFAV. One unit was added to the raw FFU values to allow log_10_ transformation of zeros. Day 0 represents the day of arbovirus inoculation, where arbovirus titers are set to 0 for visualization purposes. Arbovirus titration was performed in C6/36 cells. Each data point represents the mean for 3 (CFAV and DENV-1) or 2 (ZIKV) biological replicates, and the vertical bars are the standard errors of the means. The *P* values above the graphs were obtained from the full statistical models. Asterisks show the statistically significant differences between each treatment at each time point and the single-virus inoculation control at the same time point (*, *P* < 0.05; **, *P* < 0.01; ***, *P* < 0.001).

Using our newly isolated wild-type CFAV strain, we first performed coinfection (i.e., simultaneous infection) and superinfection (i.e., sequential infection) experiments in C6/36 cells. CFAV was inoculated onto C6/36 cells 3, 2, or 0 days prior to DENV-1 inoculation. CFAV-only, DENV-1-only, and mock inoculations of naive cells were included as controls on day 0 simultaneously with DENV-1 inoculation of CFAV-infected cells (Fig. 2A). The DENV-1 growth curve was examined by measuring infectious titers by focus-forming assay (FFA) during a 7-day time course (Fig. 2C). DENV-1 titers were significantly lower in the presence of CFAV at all time points except on day 7 in the coinfection treatment. The negative effect of CFAV on DENV-1 replication increased as the time interval between CFAV inoculation and DENV-1 inoculation increased (Fig. 2C).

We also monitored CFAV replication kinetics by reverse transcription (RT)-quantitative PCR (qPCR) on cell culture supernatants (Fig. 2B). The CFAV RNA concentration did not significantly differ between the CFAV-only control and the DENV-1–CFAV coinfection treatment at any of the time points (Fig. 2B). However, in both superinfection treatments (2-day and 3-day intervals), CFAV replication levels were significantly higher than those in the CFAV-only control and in the coinfection treatment on days 1 and 2. On day 3, CFAV replication levels were significantly higher in the superinfection treatment with a 3-day interval than in the CFAV-only and the coinfection treatments. There was no statistically significant difference among treatments on days 4 and 7 after DENV-1-only, CFAV-only, or coinfection inoculation. In addition, in the superinfection treatment with a 3-day interval, CFAV replication levels were significantly higher than those in the superinfection treatment with a 2-day interval on day 2 after DENV-1 inoculation (*P* = 0.034).

We performed another *in vitro* experiment with ZIKV, which was inoculated onto C6/36 cells 3 days after CFAV inoculation or mock inoculation, used as a control (Fig. 2D). The ZIKV growth curve was examined by measuring infectious titers by FFA during a 7-day time course (Fig. 2D). ZIKV titers were significantly lower in the presence of CFAV at late time points (4, 6, and 7 days after ZIKV inoculation). Overall, these experiments demonstrated that our wild-type CFAV strain inhibits the replicative fitness of mosquito-borne flaviviruses *in vitro*.

**FIG 3:**
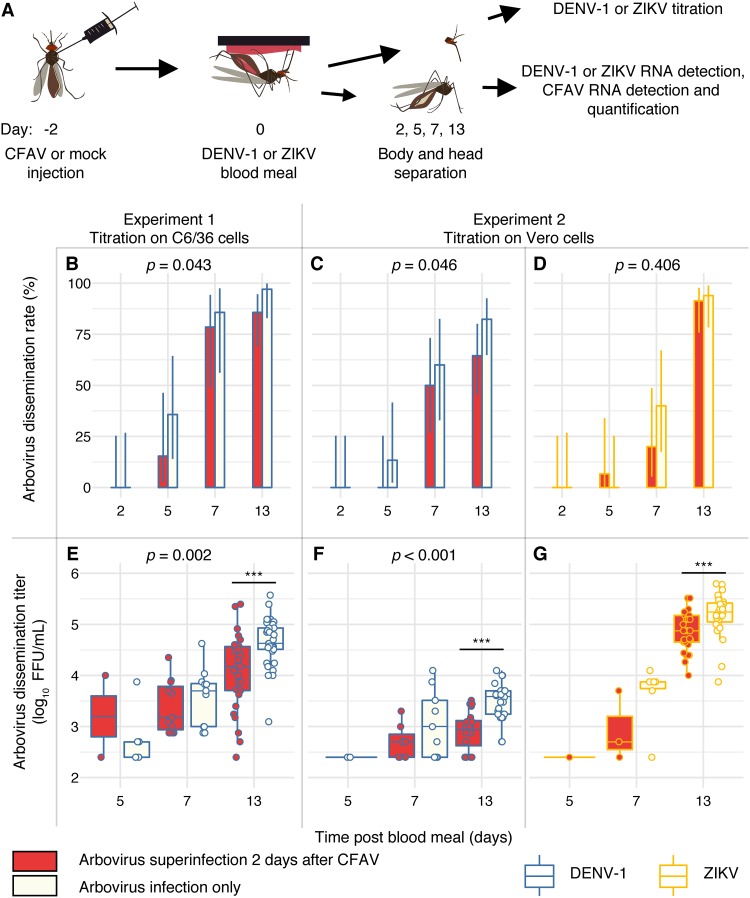
CFAV inhibits DENV-1 and ZIKV dissemination *in vivo*. (A) The schematic summarizes the design of experiments 1 and 2. Mosquitoes were orally challenged with DENV-1 or ZIKV 2 days after CFAV inoculation. Day 0 represents the day of arbovirus exposure. Arbovirus titration was performed in C6/36 (experiment 1) or Vero (experiment 2) cells. The dissemination rate is the percentage of infected mosquitoes with an arbovirus-positive head. The dissemination titer is the infectious titer per arbovirus-positive head. (B to D) Bar plots represent the dissemination rates of DENV-1 (B and C) and ZIKV (D) over time in the presence or absence of CFAV. (E to G) Box plots represent the dissemination titers of DENV-1 (E and F) and ZIKV (G) over time in log_10_-transformed focus-forming units (FFUs) per milliliter of mosquito head homogenate in the presence or absence of CFAV. The vertical bars are the 95% confidence intervals of the percentages (B to D) or 1.5 interquartile ranges (E to G). The *P* values above the graphs indicate the statistical significance of the treatment effect in a full model accounting for the time point effect (except for panel F, because the model did not meet the assumption of normal residual distribution). Asterisks show the statistically significant treatment effects at single time points (***, *P* < 0.001).

We next investigated whether inhibition of DENV-1 and ZIKV replication by our wild-type CFAV strain occurred *in vivo*. To control for virus dose and other confounding factors, we infected mosquitoes with CFAV by intrathoracic (IT) injection. Female A. aegypti mosquitoes from a CFAV-free isofemale line were intrathoracically inoculated with a standardized dose of CFAV or mock inoculated with sterile medium. Two days later, the surviving females were offered an artificial infectious blood meal containing a high dose of either arbovirus (DENV-1 or ZIKV) expected to result in close to 100% infection or a noninfectious control blood meal. Mosquitoes were subsequently collected at several time points from day 0 to day 13 post-blood meal, and their individual bodies and heads were tested separately (Fig. 3A). We carried out this *in vivo* experiment twice: the first time with DENV-1 and the second time with both DENV-1 and ZIKV.

**FIG 4:**
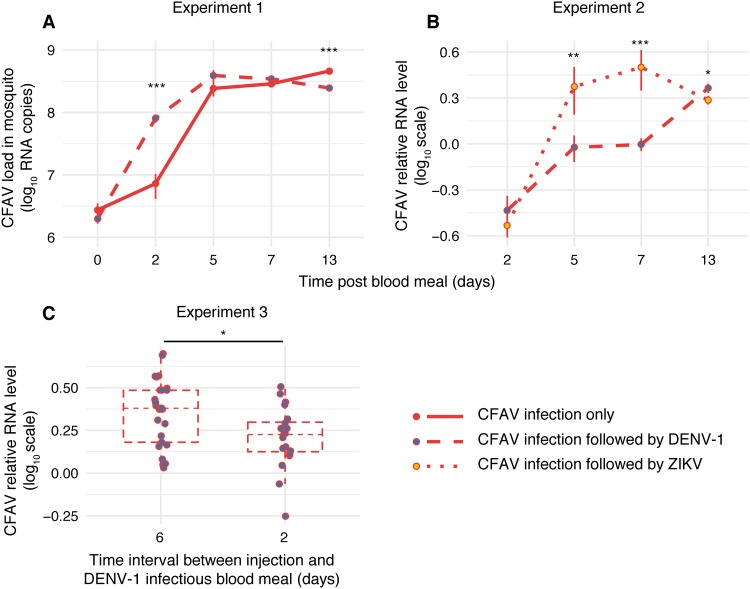
Arbovirus effects on CFAV replication *in vivo*. (A) Mosquitoes took either a DENV-1 infectious blood meal or a noninfectious blood meal 2 days after CFAV inoculation in experiment 1. (B) In experiment 2, mosquitoes took either a DENV-1 infectious blood meal or a ZIKV infectious blood meal 2 days after CFAV inoculation. (C) In experiment 3, mosquitoes took a DENV-1 infectious blood meal 6 or 2 days after CFAV inoculation. (A) Growth curves represent the CFAV load over time in log_10_-transformed viral RNA copies per mosquito body (after removing the head) in the presence or absence of DENV-1 in experiment 1. (B) Growth curves for experiment 2 represent the CFAV relative RNA level in headless bodies in the presence of DENV-1 or ZIKV. The vertical bars are the standard errors of the means. (C) Box plots for experiment 3 represent the CFAV relative RNA level in headless bodies on day 13 after a DENV-1 infectious blood meal as a function of the time interval between CFAV injection and the infectious blood meal. The vertical bars are 1.5 interquartile ranges. Asterisks show the statistically significant treatment effects at single time points (*, *P* < 0.05; **, *P* < 0.01; ***, *P* < 0.001).

The first *in vivo* experiment (referred to as experiment 1 here) consisted of 217 tested females. Out of 170 females that took a DENV-1 infectious blood meal, 164 (96%) were DENV-1 positive and processed further. The DENV-1 infection prevalence was not significantly different between CFAV-inoculated and mock-inoculated controls (98% and 95%, respectively; *P* = 0.678). In addition, 47 females that took a noninfectious blood meal were processed to assess CFAV replication in the absence of DENV-1. Across the entire time course, the proportion of DENV-1-positive mosquitoes with a DENV-1-positive head (i.e., the DENV-1 dissemination rate) was marginally significantly lower (*P* = 0.043) in the presence of CFAV (Fig. 3B). Likewise, the DENV-1 infectious titer in the DENV-1-positive heads (i.e., the DENV-1 dissemination titer) was significantly lower (*P* = 0.002) in the presence of CFAV (Fig. 3E). Pairwise comparisons at individual time points showed that this effect was primarily driven by the difference on day 13 post-blood meal (*P* < 0.001). Overall, the CFAV RNA load in mosquito bodies increased exponentially until day 5 post-blood meal (i.e., 7 days after IT inoculation) but differed significantly between DENV-1-infected and noninfected controls at two time points (Fig. 4A). At day 2 post-blood meal, the CFAV RNA load was higher in the presence of DENV-1, whereas at day 13 post-infectious blood meal, it was significantly lower than that in the no-DENV-1 control.

The second *in vivo* experiment (referred to as experiment 2 here) was designed to verify the DENV-1 results of experiment 1 and determine if they extend to ZIKV. In order to exclude a possible confounding effect of CFAV during the arbovirus titration, all focus-forming assays (FFAs) of the second experiment were performed in Vero (green monkey kidney) cells, which are mammalian and thus do not support CFAV replication. A total of 153 out of 155 females (99%) were DENV-1 positive and processed further. The DENV-1 infection prevalence was not significantly different between CFAV-inoculated and mock-inoculated controls (96% and 97%, respectively; *P* = 0.460). The two arboviruses had a different effect on CFAV replication dynamics in mosquito bodies (Fig. 4B). CFAV RNA levels were similar on day 2 post-infectious blood meal; they were significantly higher in ZIKV-infected mosquitoes on days 5 and 7 and significantly higher in DENV-1-infected mosquitoes on day 13. Across the entire time course, both the DENV-1 dissemination rate (Fig. 3C) and the DENV-1 dissemination titer (Fig. 3F) were significantly lower in the presence of CFAV. Pairwise comparisons at individual time points showed that, again, the negative effect of CFAV on the DENV-1 dissemination titer was driven by the difference on day 13 post-blood meal (*P* < 0.001). When the results of experiment 1 and experiment 2 were combined, the effect of CFAV on DENV-1 dissemination rates and titers was statistically significant (*P* = 0.005 and *P* < 0.001, respectively) without a significant interaction with the experiment effect or the time point effect.

In experiment 2, a total of 153 out of 158 females (97%) were ZIKV positive and processed further. The ZIKV infection prevalence was not significantly different between CFAV-inoculated and mock-inoculated controls (*P* = 1.0). Although there was no detectable difference in the ZIKV dissemination rate (*P* = 0.406), the ZIKV dissemination titer was significantly lower (*P* < 0.001) on day 13 post-blood meal in the presence of CFAV (Fig. 3D and G). Together, experiments 1 and 2 indicated that CFAV negatively interferes with the systemic dissemination of both DENV-1 and ZIKV in A. aegypti mosquitoes.

Because the negative controls in experiments 1 and 2 were IT inoculated with sterile cell culture medium, any other virus that would have been either (i) inadvertently coisolated or (ii) already present in the C6/36 cell line used to produce the CFAV stock could have confounded our interpretation. Indeed, the deep-sequencing data revealed that our CFAV stock produced in C6/36 cells from mosquito homogenates also contained the nearly full genomes of a putative nodavirus and Aedes albopictus densovirus 2. To determine whether these sequences reflected coisolated viruses from the mosquito homogenate or adventitious viruses commonly found in mosquito cell lines (26, 28, 30, 31), we produced a new CFAV stock directly from an RNA template. In a cDNA library prepared from the CFAV stock produced from genomic RNA and amplified in C6/36 cells, as well as in the naive C6/36 cell supernatant, we detected the putative nodavirus but not the densovirus sequences.

**FIG 5:**
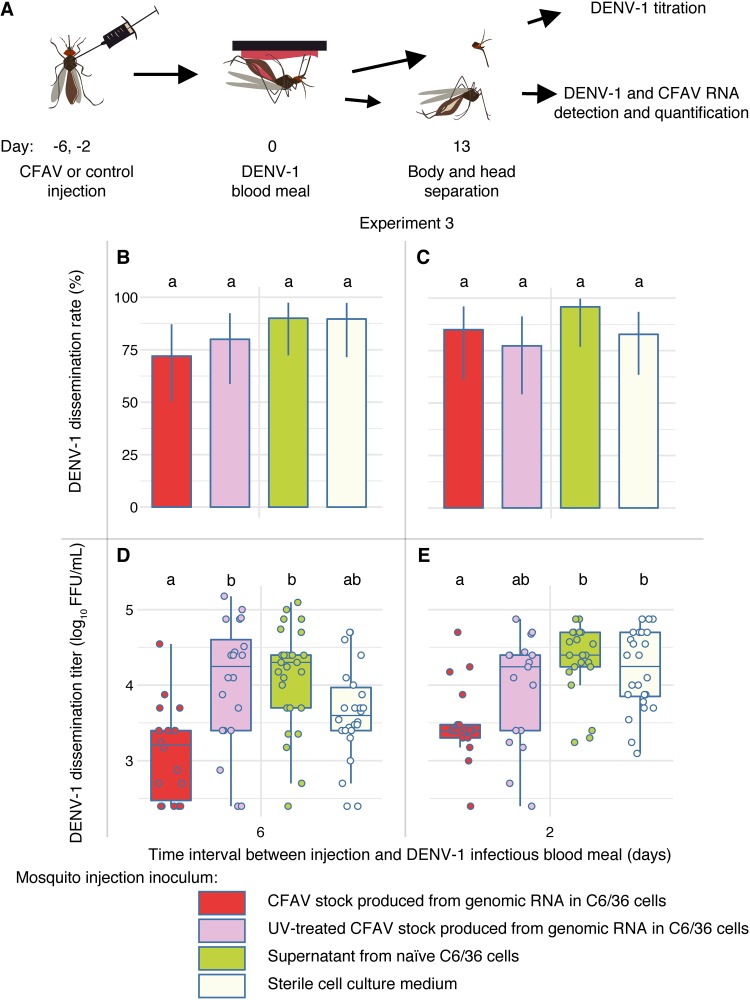
Specificity of DENV-1 inhibition by CFAV relative to potential confounding factors in the CFAV stock. (A) The schematic summarizes the design of experiment 3. Mosquitoes were orally challenged with DENV-1 at 6 days (B, D) or 2 days (C, E) after IT injection according to four treatments: a CFAV stock produced from an RNA template, a UV-treated CFAV stock produced from an RNA template, a C6/36 cell supernatant free of CFAV, and sterile L-15 cell culture medium. The dissemination rate is the percentage of infected mosquitoes with a DENV-positive head. The dissemination titer is the infectious titer in DENV-1-positive head homogenate. Dissemination rates and titers were determined by FFA titration of head homogenates in Vero cells. (B and C) The bar plots represent the DENV-1 dissemination rates on day 13 post-infectious blood meal. Vertical error bars show the 95% confidence intervals of the percentages. (D, E) The box plots represent DENV-1 dissemination titers on day 13 post-infectious blood meal. Each circle represents the log_10_-transformed number of focus-forming units (FFUs) in an individual mosquito head. Letters above the graphs represent the statistical significance of pairwise differences after correction for multiple testing. The differences between treatments with a letter in common are not statistically significantly different.

We conducted a third *in vivo* experiment (referred to as experiment 3 here) to disentangle the potential confounding effects of the putative nodavirus present in the C6/36 cells and the coisolated densovirus from the effect of CFAV. We compared the effect of the CFAV stock produced directly from genomic RNA to that of the naive C6/36 cell supernatant, UV-inactivated CFAV stock, and sterile cell culture medium as controls. The CFAV stock made from the RNA template is free of the densovirus and any other virus that could have been present in the mosquitoes of origin. Naive C6/36 cell culture supernatant presumably contains adventitious viruses also present in the CFAV stock, such as the putative nodavirus, but in the absence of CFAV. UV inactivation of this CFAV stock allows testing of the effect of inactivated virus particles or components from the cell culture supernatant. Sterile cell culture medium was the same negative control used in the previous *in vivo* experiments. Moreover, we evaluated the influence of the time interval between CFAV inoculation and arbovirus exposure on the previously observed interference phenotype. We compared the effect of CFAV injections performed 6 versus 2 days before DENV-1 exposure on the dissemination phenotype on day 13 post-blood meal (Fig. 5A).

In experiment 3, 212 out of 232 females (91%) that took a DENV-1 infectious blood meal were DENV-1 positive and processed further. No significant difference in DENV-1 infection rates was observed among treatments (*P* = 0.085). There was no detectable difference in DENV-1 dissemination rates among treatments or injection days (Fig. 5B and C). However, DENV-1 dissemination titers were significantly influenced by the day of inoculum injection (*P* = 0.005) and the treatment (*P* < 0.001). Overall, dissemination titers were significantly lower when CFAV and control inocula were injected 6 days versus 2 days prior to the infectious blood meal and lower in mosquitoes injected with the CFAV stock made from an RNA template than in those receiving the three control treatments (Fig. 5D and E). In agreement with the results of experiments 1 and 2, CFAV significantly reduced DENV dissemination titers on day 13 (*P* = 0.003) relative to those in the sterile medium injection control when CFAV was injected 2 days before the infectious blood meal. This effect was no longer statistically significant when CFAV was injected 6 days before the infectious blood meal (*P* = 0.079). CFAV also significantly reduced DENV-1 dissemination titers on day 13 (*P* < 0.001) relative to those in the C6/36 cell supernatant injection control, regardless of the day of injection. In contrast, the C6/36 cell supernatant control did not differ from the sterile medium control. Injection of a UV-inactivated CFAV stock resulted in an effect similar to that of the C6/36 cell supernatant control. The UV-inactivated CFAV stock resulted in higher DENV-1 dissemination than the CFAV stock (*P* < 0.001), but only when injection took place 6 days before the infectious blood meal. We also compared CFAV replication levels in the bodies (after removing the head) on day 13 post-infectious blood meal. The CFAV relative RNA level was higher when CFAV was injected 6 days versus 2 days before the DENV-1 blood meal (Fig. 3C). Together, experiment 3 demonstrated that the negative effect of CFAV on the DENV-1 dissemination titer on day 13 post-blood meal could not be achieved by the naive C6/36 cell supernatant (presumably containing adventitious viruses) alone, the UV-inactivated CFAV stock, or sterile medium alone.

**FIG 6:**
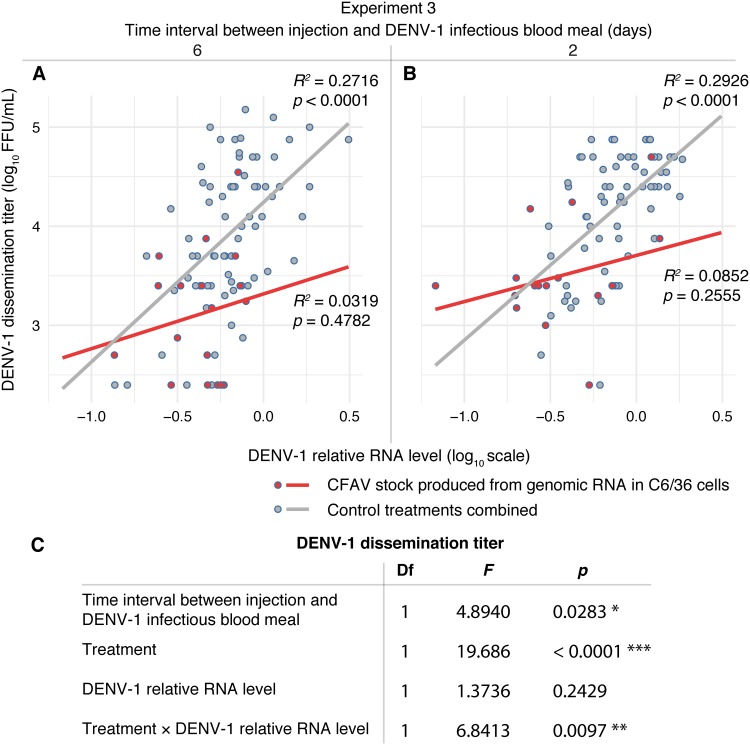
CFAV influences the relationship between DENV-1 RNA levels in bodies and DENV-1 titers in head tissues. (A and B) Scatter plots with regression lines represent the linear dependence of the log_10_-transformed DENV-1 dissemination titer (infectious titer in DENV-1-positive head homogenate) on the log_10_-transformed DENV-1 relative RNA level in the mosquito body (after removing the head) on day 13 after a DENV-1 infectious blood meal in experiment 3. CFAV was IT injected 6 (A) or 2 (B) days before the blood meal. The *R^2^* and *P* values next to the regression lines represent the goodness of fit and statistical significance of the linear relationship, respectively, for each condition. (C) The table shows the type III analysis of variance of the log_10_-transformed DENV-1 dissemination titer as the dependent variable as a function of the time interval between injection and the DENV-1 infectious blood meal (6 and 2 days), treatment (CFAV injection and all non-CFAV controls combined), the log_10_-transformed DENV-1 relative RNA level in bodies, and the interaction between the two latter variables. This analysis includes all samples with DENV-1-positive heads, with the exception of a single outlier value with an exceedingly low DENV-1 relative RNA level, which was excluded. Asterisks show the statistical significance of the effects (*, *P* < 0.05; **, *P* < 0.01; ***, *P* < 0.001). Df, degrees of freedom; *F*, *F* ratio.

**FIG 7:**
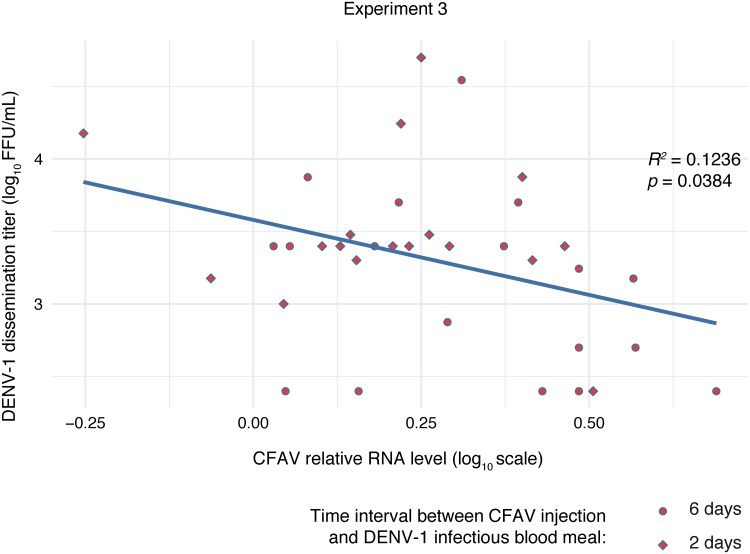
Negative linear relationship between CFAV RNA levels in the body and DENV-1 titers in head tissues. The scatter plot and regression line represent the linear dependence of the log_10_-transformed DENV-1 dissemination titer (infectious titer in DENV-1-positive head homogenate) on the log_10_-transformed CFAV relative RNA level in the mosquito body (after removing the head) on day 13 after a DENV-1 infectious blood meal in experiment 3. CFAV was IT injected 6 (circles) or 2 (diamonds) days before the blood meal. This analysis includes all samples with DENV-1-positive heads. There was no effect of the time interval between CFAV injection and the DENV-1 infectious blood meal on DENV-1 titers or RNA levels, and the time interval variable was removed from the models. *R^2^* and *P* values represent the goodness of fit and statistical significance of the linear relationship, respectively.

To explore the possible mechanisms of CFAV interference with DENV-1 systemic dissemination, we measured the relationship between DENV-1 titers in head tissues and DENV-1 relative RNA levels in headless bodies in experiment 3. Whereas the DENV-1 body RNA level was a strong linear predictor of the DENV-1 head titer in all the control treatments (combined after verifying the lack of difference between them), this was not the case in the CFAV injection treatment (Fig. 6). In order to test the dose dependence of the interference effect, we measured the linear relationship between DENV-1 titers in head tissues and CFAV relative RNA levels in headless bodies in experiment 3. We observed a negative effect of CFAV RNA levels on DENV-1 dissemination titers (Fig. 7). However, we did not detect an effect of CFAV RNA levels on DENV-1 RNA levels in bodies (*P* = 0.955), which is consistent with the previous lack of a significant relationship between DENV-1 head titers and DENV-1 body RNA levels in the presence of CFAV (Fig. 6). Together, these results indicate that CFAV reduces the systemic dissemination of infectious DENV-1 particles through the loss of a positive linear relationship between the DENV-1 RNA concentration in the body and the DENV-1 dissemination titer.

## DISCUSSION

Our results show that a newly isolated CFAV strain from Thailand is capable of inhibiting both DENV-1 and ZIKV replication in permissive mosquito cells in culture. The negative effect was stronger when CFAV infection was established for a longer time period prior to arbovirus inoculation. We also provide evidence that negative interference with DENV-1 and ZIKV occurs *in vivo* in female A. aegypti mosquitoes following IT injection of CFAV. Specifically, CFAV decreased the titer of disseminated arbovirus in the female head tissues. For DENV-1, the dissemination titer is often considered a proxy for transmission potential because it is positively correlated with virus presence in saliva (32). Although IT inoculation is not a natural mode of CFAV infection, our study provides the proof of principle that CFAV can reduce arbovirus propagation and, possibly, transmission potential in A. aegypti mosquitoes.

In addition to the negative effect of CFAV on DENV-1 replication in cell culture and dissemination in mosquitoes, we observed that DENV-1 reciprocally influenced the CFAV growth curve in A. aegypti mosquitoes following IT inoculation. However, the direction of the effect changed in the course of the infection. DENV-1 increased CFAV replication at an early time point, whereas it decreased CFAV levels at a later time point (Fig. 4A). Similar time-dependent effects were previously observed in the interaction between CFAV and DENV-2 *in vitro*. DENV-2 enhanced CFAV replication at early time points of infection, although it had no effect on CFAV replication at a late time point in A. aegypti cells (25). We also observed that DENV-1 and ZIKV differentially influenced CFAV replication kinetics in mosquito bodies (Fig. 4B). These observations suggest that ISF-arbovirus interactions are a dynamic process that may involve multiple, antagonistic effects. On the one hand, negative interference is expected when two viruses compete for the same cellular factors or when they are targeted by the same antiviral responses (20). On the other hand, upregulation of shared host factors or suppression of antiviral defense by the first virus can benefit the other virus. In the case of CFAV, the molecular mechanisms underlying the observed interference with DENV-1 and ZIKV in mosquitoes remain to be elucidated. It is also unknown how the presence of *Wolbachia* may modify this interference phenotype (16, 17).

Although the molecular mechanisms of *in vivo* interference between CFAV and arboviruses are unknown, we noticed that the positive linear relationship between the DENV-1 RNA concentration in the body and the DENV-1 dissemination titer was lost in the presence of CFAV (Fig. 6). This preliminary observation points to a lower ratio of DENV-1 infectious particles in the head tissues over DENV-1 genome copies in the body in the presence of CFAV. It supports the hypothesis that CFAV may affect arbovirus maturation and/or systemic spread rather than arbovirus genome replication *per se*. This hypothesis remains to be tested.

We used the A. albopictus cell line C6/36 rather than the common A. aegypti cell line Aag2 to assess the interaction between CFAV and DENV-1 because Aag2 cells are permanently infected with CFAV and cannot be readily compared to the appropriate CFAV-free control. Our *in vitro* results contrast with those of a previously published study that found a mutually beneficial interaction between CFAV and DENV-2 in A. aegypti cells (25). There are at least four reasons that can explain this discrepancy. First, we used a cell line from a different *Aedes* species that was previously shown to lack viral small interfering RNAs (siRNAs) that would normally activate the antiviral RNA interference (RNAi) pathway in Aag2 cells (33). An *in vitro* assay suggested that C6/36 cells exhibit inefficient cleavage of long double-stranded RNA molecules by the RNAi endoribonuclease Dicer-2 (34). This particular feature of C6/36 cells could explain the different outcome if the positive interference effect seen in A. aegypti cells was mediated by siRNAs. Second, different cell lines typically do not harbor the same adventitious viruses, which may modulate the interference phenotype. For instance, Aag2 cells are known to be persistently infected with PCLV, which is usually absent from C6/36 cells (27, 28). Third, we used a DENV-1 strain, whereas the earlier study used a DENV-2 strain. Fourth, and most importantly, we used a wild-type CFAV strain isolated from mosquitoes, whereas the earlier study used the prototype CFAV strain derived from the Aag2 cell line after decades of persistence in cell culture. The Aag2 cell-derived CFAV strain is genetically divergent from natural CFAV isolates (11) and includes potentially important functional changes, such as premature stop codons in the *fifo* −1 open reading frame (29). Phylogenetically, our CFAV strain clusters with another CFAV sequence detected in Thai mosquitoes, whereas CFAV sequences derived from the Aag2 cell line constitute a distinct clade from all other known CFAV strains (Fig. 1). Overall, our experiments *in vivo* with a wild-type CFAV strain did not recapitulate the observations previously made *in vitro* with the Aag2 cell-derived CFAV strain.

To test our *in vitro* results in a more biologically relevant situation, we IT inoculated our wild-type CFAV strain from Thailand into A. aegypti females originating from the same area where the virus was isolated and assessed their vector competence for DENV-1 and ZIKV. IT inoculation is not a natural mode of CFAV infection, but it allows accurate control of virus dose and other potentially confounding factors, such as the mosquito genotype. We found that CFAV infection significantly reduced arbovirus dissemination to the mosquito head tissues. The effect was remarkably consistent across experiments and arboviruses (Fig. 3 and 5). Importantly, we ruled out the potential confounding effect of other viruses present in the CFAV stock due to inadvertent coisolation and/or a persistent infection in the C6/36 cell line used to isolate and amplify CFAV (Fig. 5). Although the interference effect was relatively modest, it may have meaningful consequences for arbovirus transmission because the amount of disseminated DENV is a significant predictor of the probability of detection of infectious virus in salivary secretions (32). Additional work is required to determine whether the differences in dissemination titers observed in our experiments actually translate into differences in arbovirus transmission potential. Finally, our results may have implications for vector competence studies in the laboratory because the undetected presence of insect-only viruses in field samples or mosquito colonies could confound the results of vector competence assays.

Our study adds to the growing evidence that ISFs can interfere with arbovirus transmission by mosquitoes, which is reviewed extensively elsewhere (4, 15, 18–21). The current literature consists of largely inconsistent results on the magnitude and direction of ISF-arbovirus interference, which may reflect the large variety of virus strains, mosquito strains, phenotypes, and experimental methods. Because we used a wild-type CFAV strain, sympatric mosquitoes, and vector competence assays *in vivo* for two different arboviruses, our study provides rare data in support of a negative effect of ISF infection on arbovirus dissemination in mosquitoes. It calls for further investigations on the mechanistic basis of this interference phenotype. Ultimately, such investigations will support the proposed use of ISFs as biocontrol agents against arboviruses (15) and elucidate the complex interactions that occur when several viruses share the same host.

## MATERIALS AND METHODS

### Ethics statement.

This study used human blood samples to prepare mosquito artificial infectious blood meals. Healthy donor recruitment was organized by the local investigator assessment using medical history, laboratory results, and clinical examinations. Biological samples were supplied through participation of healthy volunteers at the ICAReB biobanking platform (BB-0033-00062/ICAReB platform/Institut Pasteur, Paris, France/BBMRI AO203/[Bioresource]) of the Institut Pasteur to the CoSImmGen and Diagmicoll protocols, which have been approved by the French Ethical Committee Ile-de-France I. The Diagmicoll protocol was declared to the French Research Ministry under reference DC 2008-68 COL 1.

### Viruses.

**(i) CFAV isolation.** A wild-type CFAV strain was isolated from the 4th generation of a laboratory colony of A. aegypti mosquitoes originally sampled in 2013 from a wild population in Thep Na Korn Village, Kamphaeng Phet Province, Thailand. Adult mosquitoes were homogenized in pools of 9 females in 1 ml of Leibovitz’s L-15 medium (Gibco Thermo Fisher Scientific). Homogenates were clarified by two rounds of centrifugation, and supernatants were filtered through 0.2-μm-pore-size filters (Minisart; Merck) to remove bacteria. Subconfluent C6/36 cells in 25-cm^2^ flasks were inoculated with 500 μl of the filtered homogenate and incubated at 28°C. After 1 h of incubation, 7 ml of Leibovitz’s L-15 medium complemented with 2% fetal bovine serum (FBS; Gibco Thermo Fisher Scientific), 2% tryptose phosphate broth (TPB; Gibco Thermo Fisher Scientific), 1× nonessential amino acids (NAA; Gibco Thermo Fisher Scientific), 10 U/ml of penicillin (Gibco Thermo Fisher Scientific), and 10 μg/ml of streptomycin (Gibco Thermo Fisher Scientific) was added to the flask. After 7 days of virus amplification, the cell culture supernatant was harvested and aliquoted with 10% FBS. The pH was adjusted with 0.075% sodium bicarbonate, and the virus stock was stored at −80°C. The CFAV isolate was subsequently passaged three times following the same procedure described above to produce a virus stock.

**(ii) Production of CFAV from genomic RNA.** Total RNA was extracted from the CFAV stock using a QIAamp viral RNA minikit (Qiagen) according to the manufacturer’s instructions. Reverse transcription was performed using SuperScript IV reverse transcriptase (Thermo Fisher Scientific) following the manufacturer’s guidelines and a reverse primer located at the 3′ end of the CFAV genome (Table 1). DNA amplification was performed using Q5 high-fidelity DNA polymerase (New England Biolabs) according to the manufacturer’s instructions with primers located at both extremities of the CFAV genome (Table 1). T7 *in vitro* transcription was performed with a mMESSAGE mMACHINE kit (Ambion by Life Technologies) according to the manufacturer’s instructions to produce large quantities of RNA from full-genome PCR amplicons. On the day before RNA transfection, 12.5-cm^2^ flasks were seeded with C6/36 cells to allow approximately 80% confluence the next day. Three micrograms of RNA was added to 300 μl of Opti-MEM medium (Gibco Thermo Fisher Scientific), 6 μl of mRNA boost reagent (Mirus Bio), and 6 μl of TransIT mRNA reagent (Mirus Bio) before a 3-min incubation at room temperature (20 to 25°C). The cells were incubated with the transfection mixture for 4 h at 28°C. At the end of the incubation, the transfection mixture was removed and replaced by Leibovitz’s L-15 medium with 2% FBS, 2% TPB, and 1× NAA. At 7 days posttransfection, cell culture supernatants were harvested and amplified twice in C6/36 cells with 7-day passages. The presence of CFAV was assessed by RT-PCR as well as observation of a CFAV-characteristic CPE, namely, fusion of cells.

**TABLE 1:** Primers used for RT-PCR detection and RT-qPCR quantification of DENV-1, ZIKV, CFAV, and Aedes aegypti housekeeping genes *rp49* and *rps7*

Genome	Genomic region	Application	Direction	Primer sequence (5′ → 3′)	Length of PCR product (bp)	Annealing temp (°C)
DENV-1	NS5	Detection	Forward	GGAAGGAGAAGGACTCCACA	105	60
DENV-1	NS5	Detection	Reverse	ATCCTTGTATCCCATCCGGCT	105	60
ZIKV	NS1	Detection	Forward	GTATGGAATGGAGATAAGGCCCA	191	60
ZIKV	NS2A	Detection	Reverse	ACCAGCACTGCCATTGATGTGC	191	60
CFAV	NS3	Quantification	Forward	ACACGAGTGAAGCTGGTTGA	92	56
CFAV	NS3	Quantification	Reverse	ACATACGTTCCTGGTTCCCG	92	56
CFAV	E	Detection	Forward	GCTTCAAGTGGGGGATTGGA	345	62
CFAV	E	Detection	Reverse	CAACTTTCTCCATGCCGTGC	345	62
CFAV	5′ UTR^*a*^	Genomic cDNA amplification	Forward	AGTTTATAAAAACTTCGGCTTGGCT	10,696	60
CFAV	3′ UTR	RT, genomic cDNA amplification	Reverse	AGCGCATCTATGGTATAGAAAAGA	10,696	60
Aedes aegypti	*rp49*	Housekeeping gene quantification	Forward	ACAAGCTTGCCCCCAACT	97	60
Aedes aegypti	*rp49*	Housekeeping gene quantification	Reverse	CCGTAACCGATGTTTGGC	97	60
Aedes aegypti	*rps7*	Housekeeping gene detection	Forward	GGGACAAATCGGCCAGGCTATC	292	64
Aedes aegypti	*rps7*	Housekeeping gene detection	Reverse	TCGTGGACGCTTCTGCTTGTTG	292	64

a UTR, untranslated region.

**(iii) CFAV titration.** The infectious titer of the CFAV stocks (both the original isolate and the virus produced from genomic RNA) was determined by 50% tissue culture infectious dose (TCID_50_) assay in C6/36 (A. albopictus) cells using the CFAV-characteristic CPE as an indication of CFAV infection. On day −1, 96-well plates with a flat bottom were seeded with 100 μl at 2 × 10^6^ cells/ml. On day 0, the cell layer reached 70 to 90% confluence and the medium was removed. Each well was inoculated with 40 μl of sample in serial 10-fold dilutions with multiple replicates (>4) per sample. The cells were incubated for 1 h at 28°C under atmospheric CO_2_. At the end of the incubation, 60 μl of fresh Leibovitz’s L-15 medium complemented with 2% FBS, 2% TPB, 1× NAA, 10 U/ml of penicillin, and 10 μg/ml of streptomycin was added to each well. The plates were sealed with autoclave tape to prevent contamination and incubated at 28°C under 5% CO_2_. After 6 days of incubation, the cell culture medium was replaced with fresh medium and the plates were incubated for another 3 to 5 days until a CPE became clearly distinguishable. The proportion of wells with cell fusion was counted for each dilution level, and the infectious titer was calculated using the method of Reed and Muench to estimate 50% endpoints (35). The titer of the original CFAV stock was 1.14 × 10^7^ TCID_50_ units/ml. The titer of the CFAV stock produced from genomic RNA was 5.39 × 10^6^ TCID_50_ units/ml.

**(iv) UV inactivation of CFAV produced from genomic RNA.** To inactivate the virus, 1 ml of CFAV stock was spread on a sterile petri dish and exposed to a dose of 2 J/cm^2^ of 254-nm UV irradiation in a CL-1000 UV cross-linker (UVP). Inactivation was verified by detecting viral RNA in IT-inoculated mosquitoes by RT-PCR using CFAV detection primers (Table 1). Only 4 mosquitoes were found to be CFAV positive after IT inoculation of the UV-inactivated CFAV stock and were removed from further analysis.

**(v) Arboviruses.** The DENV-1 strain used in this study (KDH0026A, GenBank accession number HG316481) was originally isolated in 2010 from the serum of a patient attending Kamphaeng Phet Provincial Hospital, Thailand (36). The ZIKV strain used in this study (PF13/251013-18, GenBank accession number KY766069) was isolated at the Institut Louis Malardé in 2013 from the serum of a patient in French Polynesia (37). Both viruses were amplified on C6/36 cells in order to generate high-titer stocks as described previously for DENV (38). The infectious titer in C6/36 cells (*in vitro* experiments and the first *in vivo* experiment) or Vero cells (the second and third *in vivo* experiments) was measured by a standard focus-forming assay (FFA) as previously described for DENV in C6/36 cells (38). The FFA in Vero cells is similar to the FFA in C6/36 cells, except that incubation lasts for 3 days for DENV and 2 days for ZIKV. The primary antibody was a mouse anti-flavivirus group antigen monoclonal antibody (catalog number MAB10216; Merck Millipore) diluted 1:1,000 for ZIKV and a mouse anti-DENV complex monoclonal antibody (catalog number MAB8705; Merck Millipore) diluted 1:200.

### CFAV sequencing and phylogenetic analysis.

**(i) Library preparation and deep sequencing.** For preparation of cDNA libraries from the original CFAV stock, the CFAV stock produced from genomic RNA, and the naive C6/36 cell supernatant, RNA was extracted using a QIAamp viral RNA minikit (Qiagen) according to the manufacturer’s instructions and eluted in 60 μl of AE buffer (Qiagen). RNA was subsequently treated with 4 units of Turbo DNase (Ambion), and cDNA was produced using Moloney murine leukemia virus (M-MLV) reverse transcriptase (Invitrogen) following the manufacturer’s instructions. Twenty microliters of cDNA was incubated for 2 h at 16°C with 10 units of Escherichia coli DNA ligase (New England Biolabs), 40 units of E. coli DNA polymerase I (New England Biolabs), and 2 units of E. coli RNase H (New England Biolabs) for second-strand synthesis with second-strand synthesis buffer (New England Biolabs) on 20 μl of single-stranded DNA. Purified double-stranded DNA was used for library preparation using a Nextera XT DNA kit (Illumina) according to the manufacturer’s instructions, followed by a cDNA quality check by use of a Bioanalyzer DNA 1000 kit (Agilent). The libraries were combined with other libraries from unrelated projects and loaded onto a NextSeq 500/550 mid-output kit (v2) 150-cycle flow cell (Illumina). Sequencing was performed on paired ends on an Illumina NextSeq 500 instrument. Raw sequencing data sets were deposited in the SRA database.

**(ii) Sequencing data analysis.** The sequencing data were processed following a pipeline described elsewhere (39). Briefly, sequencing reads with a quality score of <30 were trimmed using the Trimmomatic (v0.36) tool (40). CFAV reads were mapped to the Aedes albopictus genome using the Bowtie2 (v2.3.4.3) program, and the remaining reads were subjected to *de novo* assembly with the Ray (v2.3.1-mpi) tool (41, 42). Scaffolds were subjected to a blastn search for similarities in the nucleotide database using BLAST+ (v2.2.40) software (43). Output blastn hits were filtered for only viral hits by use of the keyword “virus” in the GenBank file. A preliminary consensus CFAV genome sequence was constructed by mapping with the Bowtie2 (v2.3.4.3) program. To detect adventitious viruses, unaligned reads were *de novo* assembled into contigs and scaffolds and subjected to a blastn search. Almost full genomes of two viruses other than CFAV were detected in the cDNA library prepared from the original CFAV stock derived from mosquito homogenate. These viruses were a putative nodavirus closely related to Flock House virus and Drosophila melanogaster American nodavirus and Aedes albopictus densovirus 2. The putative nodavirus, but not the densovirus, was detected in the cDNA library prepared from the CFAV stock produced from genomic RNA. Traces of Culex Y virus and Megavirus vitis were also detected in both cDNA libraries, although it was not possible to assemble their genomes. DENV was detected in the CFAV stock produced from mosquito homogenates and the naive C6/36 cell supernatant, but examination of the sequence revealed that it resulted from cross-contamination from DENV libraries that were simultaneously sequenced.

**(iii) Sequence verification at the extremities.** The extremities of the consensus CFAV genome sequence were verified by rapid amplification of cDNA ends (RACE) using a 5′/3′ RACE kit, 2nd generation (Roche), following the manufacturer’s instructions with prior poly(A) addition to the 3′ end of RNA using E. coli poly(A) polymerase (New England Biolabs). The final CFAV full-genome sequence was deposited in the European Nucleotide Archive.

**(iv) Phylogenetic analysis.** All available full or nearly full genome sequences of CFAV were retrieved from GenBank (accession numbers KJ476731, GQ165810, KJ741267, MH310082, KU936054, MH237596, and M91671) or may be obtained from the authors upon request (CFAV_Australia_2014 and CFAV_Thailand_2015) (12). All CFAV sequences were aligned using MAFFT nucleotide alignment under default settings in the Geneious (v10.2.3) program (44). A GTR+F+I substitution model was selected based on the best Bayesian information criterion score by the ModelFinder program, followed by maximum likelihood tree reconstruction in the IQ-TREE (v1.6.3) program (45, 46).

### Mosquitoes and cell lines.

**(i) Mosquitoes.** An isofemale line of A. aegypti originating from Kamphaeng Phet Province, Thailand, was used for experimental infections *in vivo*. The isofemale line was created in 2010 as the progeny of a single-pair mating between a wild male from Mae Na Ree Village and a wild female from Nhong Ping Kai Village (36). The isofemale line was maintained under standard insectary conditions (27°C, 70% relative humidity, and a 12-h light and 12-h dark cycle) for 25 generations prior to its use in this study. It was confirmed to be free from CFAV by failure to isolate the virus on C6/36 cells from mosquito homogenates and a negative result by RT-PCR directly on mosquito RNA. Prior to experimental infections, larvae were reared in plastic trays filled with 1.5 liters of dechlorinated tap water at a density of 200 larvae per tray and provided with 200 mg of TetraMin fish food (Tetra) on days 0 and 2 and 400 mg on day 4. After emergence, adult mosquitoes were housed in plastic cages under standard insectary conditions (27°C, 70% relative humidity, and a 12-h light and 12-h dark cycle) and provided with 10% sucrose solution *ad libitum*.

**(ii) Cell lines.** The Aedes albopictus cell line C6/36 (ATCC CRL-1660) was used for amplification of all virus stocks, experimental infections *in vitro*, and titration assays. C6/36 cells were maintained at 28°C under atmospheric CO_2_ in flasks with nonvented caps in Leibovitz’s L-15 medium containing 10% FBS, 2% TPB, 1× NAA, 10 U/ml of penicillin, and 10 μg/ml of streptomycin. The Cercopithecus aethiops cell line Vero (ATCC CCL-81) was used in titration assays. Vero cells were maintained at 37°C under 5% CO_2_ in Dulbecco’s modified Eagle medium (DMEM; high glucose; GlutaMAX supplement; Gibco Thermo Fisher Scientific) without sodium pyruvate provided with 10% FBS, 10 U/ml of penicillin, and 10 μg/ml of streptomycin.

### Experimental infections *in vitro*.

C6/36 cells were grown in 6-well plates with 5 ml/well of Leibovitz’s L-15 medium with 10% FBS, 2% TPB, 1× NAA, 10 U/ml of penicillin, and 10 μg/ml of streptomycin. Subconfluent cells were inoculated with CFAV at a multiplicity of infection (MOI) of 0.23. After CFAV infection, cells were maintained in 5 ml/well of Leibovitz’s L-15 medium with 2% FBS, 2% TPB, 1× NAA, 10 U/ml of penicillin, and 10 μg/ml of streptomycin. Three, 2, or 0 days after CFAV infection, the cells were inoculated with DENV-1 at an MOI of 0.1. Cells with no prior CFAV infection were used as a negative control. Aliquots of cell culture supernatant of 150 μl were collected on days 1, 2, 3, 4, and 7 after DENV-1 infection, from which 40 μl was used for DENV-1 titration and 100 μl was used for RNA extraction and RT-qPCR. The ZIKV superinfection experiment followed the same procedure, except that the CFAV MOI was 0.11, the cells were grown in 75-cm^2^ flasks with 20 ml of medium, ZIKV was only inoculated 3 days after CFAV infection, and supernatant collections were done on days 1, 2, 3, 4, 6, and 7 after ZIKV infection.

### Experimental infections *in vivo*.

In two independent experiments, 262 TCID_50_ units of CFAV diluted in sterile Leibovitz’s L-15 medium with 2% TPB, 1× NAA, 10 U/ml of penicillin, and 10 μg/ml of streptomycin or the same sterile medium alone (mock injection) were injected into the thorax of 2-day-old (first and second *in vivo* experiments) or 2-day-old and 6-day-old (third *in vivo* experiment) A. aegypti females. IT injections were performed using a Nanoject II auto-nanoliter injector (Drummond) in the first *in vivo* experiment and a Nanoject III programmable nanoliter injector (Drummond) in the second and third *in vivo* experiments. Mosquitoes from each treatment were distributed into three 1-pint cylindrical cardboard boxes with a fine mash on top and maintained under standard insectary conditions with 10% sucrose solution *ad libitum*. Two days after the IT injection, mosquitoes were offered a DENV-1 or ZIKV infectious blood meal containing 5 × 10^6^ focus-forming units (FFU)/ml and 7.5 × 10^6^ FFU/ml, respectively. To prepare the artificial infectious blood meal, the arbovirus stock was diluted in sterile growth medium (Leibovitz’s L-15 medium with 10% FBS, 2% TPB, 1× NAA, 10 U/ml of penicillin, 10 μg/ml of streptomycin, and 0.075% sodium bicarbonate) and mixed 1:2 with freshly drawn human erythrocytes that had been washed and diluted in sterile distilled phosphate-buffered saline (DPBS; Gibco Thermo Fisher Scientific). After gentle mixing, 2.5 ml of the infectious blood meal was placed into each of several Hemotek membrane feeders (Hemotek Ltd.) maintained at 37°C and covered with a piece of desalted porcine intestine as a membrane. Sixty microliters of 0.5 M ATP was added to each feeder as a phagostimulant. Mosquitoes deprived of water and the sucrose source for 18 h were offered the infectious blood meal for 15 min. Fully blood-fed females were sorted and incubated in climatic chambers at 27°C and under 70% relative humidity and a 12-h light and 12-h dark cycle. Mosquitoes were harvested on days 0, 2, 5, 7, and 13 post-infectious blood meal. They were cold anesthetized, and the head and body from each mosquito were separated from each other. Individual heads were homogenized in 300 μl of Leibovitz’s L-15 medium with 2% TPB, 1× NAA, 10 U/ml of penicillin, and 10 μg/ml of streptomycin and stored at −80°C until further use in the first two *in vivo* experiments (experiments 1 and 2). In the third *in vivo* experiment (experiment 3), the heads were initially stored at −80°C and homogenized before arbovirus titration. Individual bodies were homogenized in 400 μl of lysis buffer for RNA extraction (RAV1; NucleoSpin virus core kit; Macherey-Nagel) and stored at −80°C until further use. The bodies were used to determine the arbovirus infection rate by RT-PCR (experiments 1 and 2), DENV-1 relative RNA levels (experiment 3), CFAV loads and relative RNA levels by RT-qPCR (experiments 1 and 2, respectively), and CFAV infection status by RT-PCR and CFAV relative RNA levels (experiment 3). The primer sequences are provided in Table 1. Heads from arbovirus-positive mosquitoes were used to determine the arbovirus dissemination rate and titer by FFA as described above.

### RNA extraction and RT-PCR.

Total RNA was extracted and purified from cell culture supernatants or mosquito bodies using the NucleoSpin virus core kit (Macherey-Nagel) following the manufacturer’s instructions with RNA elution in 50 μl of RNase-free water at 70°C for bodies and 100 μl for cell culture supernatants. cDNA synthesis was performed using M-MLV reverse transcriptase (Invitrogen) by mixing 5 μl of eluted RNA with 100 ng of random primers (Roche), 10 nmol of each deoxynucleoside triphosphate, 2 μl of dithiothreitol, 4 μl of 5× first-strand buffer, 5.5 μl of PCR-grade water, 20 units of RNaseOUT recombinant RNase inhibitor (Invitrogen), and 200 units of M-MLV reverse transcriptase in a final reaction volume of 20 μl. The reaction mixtures were incubated for 10 min at 25°C, 50 min at 37°C, and 15 min at 70°C and held at 4°C until further use or stored at −20°C. Diagnostic PCRs for all three viruses was performed with DreamTaq Green DNA polymerase (Thermo Scientific) following the manufacturer’s recommendations. Quantitative analysis by qPCR was done using GoTaq qPCR master mix (Promega) following the manufacturer’s recommendations. The primer sequences are provided in Table 1.

### Statistical analyses.

Statistical analyses were performed in the statistical environment R (v3.5.2; http://www.r-project.org/). In all analyses, the individual mosquito sample was considered a biological unit of replication. Continuous variables (infectious titers and RNA loads) were analyzed by type III multivariate analysis of variance (MANOVA) of log_10_-transformed values. Binary variables (infection and dissemination status) were analyzed by logistic regression and type III analysis of deviance. The interaction term was removed from the model when it was statistically insignificant in the full model (*P* > 0.05). When the interaction was removed from the model, continuous variables were analyzed by type II MANOVA and binary variables were analyzed by type II analysis of deviance. Time point was considered an ordinal variable, and injection day was considered a nominal variable. Infection and dissemination rates were compared between each pair of treatments at each time point by a pairwise Pearson χ^2^ test. Infectious titers and RNA loads were compared between each pair of treatments by Welch’s *t* test or by the Wilcoxon signed-rank test when the residual distribution did not meet the normality assumption. When more than two treatments were compared, *P* values were adjusted for multiple testing using the Bonferroni correction.

### Data availability.

Raw sequencing data sets were deposited in the SRA database (CFAV stock, accession number SRR8996077; CFAV stock produced from genomic RNA, accession number SRR8996076; naive C6/36 cell supernatant, accession number SRR8996075). The final CFAV full-genome sequence was deposited in the European Nucleotide Archive (accession number LR596014).
